# Estimating the Impacts of Plant Internal Nitrogen Deficit at Key Top Dressing Stages on Corn Productivity and Intercepted Photosynthetic Active Radiation

**DOI:** 10.3389/fpls.2022.864258

**Published:** 2022-04-08

**Authors:** Ben Zhao, Syed Tahir Ata-Ul-Karim, Aiwang Duan, Yang Gao, He Lou, Zugui Liu, Anzhen Qin, Dongfeng Ning, Shoutian Ma, Zhandong Liu

**Affiliations:** ^1^Key Laboratory of Crop Water Use and Regulation, Ministry of Agriculture, Farmland Irrigation Research Institute, Chinese Academy of Agricultural Sciences, Xinxiang, China; ^2^Graduate School of Agricultural and Life Sciences, The University of Tokyo, Tokyo, Japan; ^3^Henan Weisheng Electric Limited Company, Xinxiang, China

**Keywords:** corn, nitrogen, nitrogen diagnosis index, critical nitrogen concentration, plant growth status

## Abstract

Accurate and timely appraisal of plant nitrogen (N) demand is imperative to regulate the canopy structure and corn production. The strength and time of plant N deficit can be quantified by critical N concentration. The study was aimed to analyze nitrogen nutrition index (NNI), nitrogen deficit content (NDC), plant nitrogen productivity (PNP), and a fraction of intercepted photosynthetic active radiation (FIPAR) across different N treatments and to develop NNI-NDC, NNI-PNP, NNI-FIPAR, NDC-PNP, and NDC-FIPAR relationships from V6 to V12 stages of corn to quantify the suitable PNP and FIPAR values under the optimal plant N condition. Four multi-N rates (0, 75, 90, 150, 180, 225, 270, and 300 kg N ha^−1^) field experiments were conducted with two cultivars of corn in Henan province of China. Results indicated that N fertilization affected yield, plant biomass, plant N content, and leaf area index. The values of NNI and NDC were from 0.54 to 1.28 kg ha^−1^ and from −28.13 to 21.99 kg ha^−1^ under the different treatments of N rate, respectively. The NDC and NNI showed significantly negative relationships from V6 to V12 stages. The values of PNP and FIPAR increased gradually with the crop growth process. The PNP values gradually declined while the FIPAR values of every leaf layer increased with the increase of N supply. The NDC-PNP and NNI-FIPAR relationships were significantly positive; however, the relationships between NNI-PNP and NDC-FIPAR were significantly negative during the vegetative period of corn. The coefficient of determination (*R*^2^) based on NNI was better than that on NDC. The FIPAR values were ~0.35, 0.67, and 0.76% at the upper, middle, and bottom of leaf layers, respectively, and PNP values were ~39, 44, and 51 kg kg^−1^ at V6, V9, and V12 stages, respectively, when NNI and NDC values were equal to 1 and 0 kg ha^−1^, respectively. This study described the quantitative information about the effect of a plant's internal N deficit on plant N productivity and canopy light intercept. The projected results would assist in predicting the appropriate plant growth status during key N top-dressing stages of corn, which can optimize N application and improve N use efficiency.

## Introduction

Corn is an important cereal crop, and its grain can be used for human consumption and as a feedstock (Hammad et al., 2018). Corn production in China increased tremendously during the past few decades due to the development of high-yielding cultivars and the over application of nitrogen (N) fertilizer (Cui et al., 2008). N is the most important nutrition element known to limit crop yield and quality after water deficit (Zheng et al., 2017). The Chinese farmers often applied the maximum N fertilizer amount to assure plant growth and to get a high grain yield. Over N application (average 205 kg N ha^−1^ season^−1^) has caused poor N use efficiency in the major corn planting regions of China, which has been reported to be critically dropped to 26% as compared to other developed countries and regions where it is 40–50% (Omara et al., 2019; Qi and Pan, 2022). Conversely, excessive N application is leading to multiple side effects perturbing aquatic and terrestrial ecosystems (Geary et al., 2020). Crop and environmental scientists are trying to overcome the economic and ecological problems associated with the excessive N fertilizer by developing the N management strategies, which can cut the N application rate to minimize pollution risks without affecting the grain yield (Zhao et al., 2020). Islam et al. (2012) showed that the intensity and time of N application have a synergistic function to increase harvested yield and N use efficiency. Corn development and grain yield varied with location and season response to N fertilizer (Dhital and Raun, 2016). This variation brings some difficulties to determine the crop N status during the corn growth under different environments. At present, it lacks a good way to understand crop N status and its production capacity during corn growth in China.

Non-destructive diagnostic techniques (sensors, chlorophyll meter, and digital camera) were used to crop N management. However, these techniques were different in range, spatial scale, and time resources (Ata-Ul-Karim et al., 2016a,b), and their accuracy is affected by abiotic stresses, canopy characteristics, soil background, sensor viewing geometry, and solar illumination (Ata-Ul-Karim et al., 2017a,b). The critical N concentration (N_c_) was widely used as a classical technique to diagnose the N status of various crop species (Justes et al., 1994), which has a simple, biologically sound, and specific characteristic. The critical curve constructed by Plénet and Lemaire (2000) is considered as a reference corn curve (%N_c_ = 3.4DM^−0.37^ when DM ≥ 1 t ha^−1^; %N_c_ = 3.4% when DM <1 t ha^−1^) and has been successfully tested for its applicability in corn-growing regions of China (Liang et al., 2013). Several studies have been reported by using the N_c_ curve-based N nutrition index (NNI) for the diagnosis of plant N status (Lemaire and Gastal, 1997; Liang et al., 2013; Zhao et al., 2017). Additionally, the NNI has also been used to estimate harvested yield, quality, leaf area index, and photosynthetic active radiation (PAR) (Bélanger et al., 1992; Hu et al., 2014). However, NNI cannot quantify the real value of crop N deficit. Therefore, an alternative diagnosis index based on crop N_c_ concentration has been derived to quantitatively represent the real value of plant N deficit in crops (Yao et al., 2014a,b), which is named as N deficit content (NDC) (Ata-Ul-Karim et al., 2013). So far, NDC has not yet been used to describe plant N deficit and growth indices of corn quantitatively.

The optimal N management requires the knowledge of plant N deficit during crop vegetative growth, which can induce the reasonable plant N productivity (PNP) in this period (Hu et al., 2014). The PNP was introduced to interpret the dependency of plant growth on internal N, which is described as the increase of plant biomass per unit of plant N content (Lemaire et al., 2008). The change of PNP was affected by the external N environment and plant internal N deficit (Lemaire and Gastal, 1997). Greenwood et al. (1991) showed that the PNP can keep constant value during the vegetative period of crop growth under non-limiting N conditions. On the other hand, the production efficiency of plant biomass is also related to the fraction of intercepted photosynthetic active radiation (FIPAR) within the canopy during crop growth (Zhang et al., 2008). The plant N deficit can affect the structure of the canopy and the distribution of FIPAR within the canopy. The FIPAR values showed distribution differences from top to bottom of canopy height, which can induce the optimized vertical N distribution in the canopy for maximized photosynthesis (Hirose and Werger, 1987). At present, most studies focused on the effect of external N input on the canopy distribution of FIPAR at the crop vegetative stage (Campbell et al., 2001; Ruiz and Bertero, 2008). Hernández et al. (2015) showed that the FIRAR of corn canopy increased in response to external N (0–120 kg ha^−1^) supply both under irrigated and rain-fed conditions. However, the external N input has some limitations to evaluate canopy light interception and PNP, due to the unstable relationship between external N input and plant canopy structure. Its parameters were affected by years, sites, or N treatments (Prieto et al., 2012).

Plant internal N deficit has been evaluated by crop N_c_ curve (Zhao et al., 2017). The development of the crop N_c_ curve based on crop N dilution theory has a close relationship with the physiological process occurring in the plant during its growth period (Lemaire et al., 2008). We hypothesized that plant internal N deficit could better respond to the change of crop canopy structure and production capacity under different N conditions. Louarn et al. (2015) combined light attenuation and NNI to develop the empirical model of alfalfa canopy N distribution. At present, the simulation of canopy N distribution on corn has received comparatively less attention. Little information has been found about the connection between plant N productivity, canopy structure, and plant internal N deficit during the vegetative period of corn by using the N_c_ curve. Therefore, the main objectives of this study were to describe the changes in NNI, NDC, PNP, and FIPAR across different N treatments and to analyze the effect of crop internal N deficit on PNP and FIPAR of corn vegetative growth. It is useful to explore the reasonable and efficient development of canopy structures to improve plant production efficiency on corn.

## Materials and Methods

### Experimental Design

**Figure 1:**
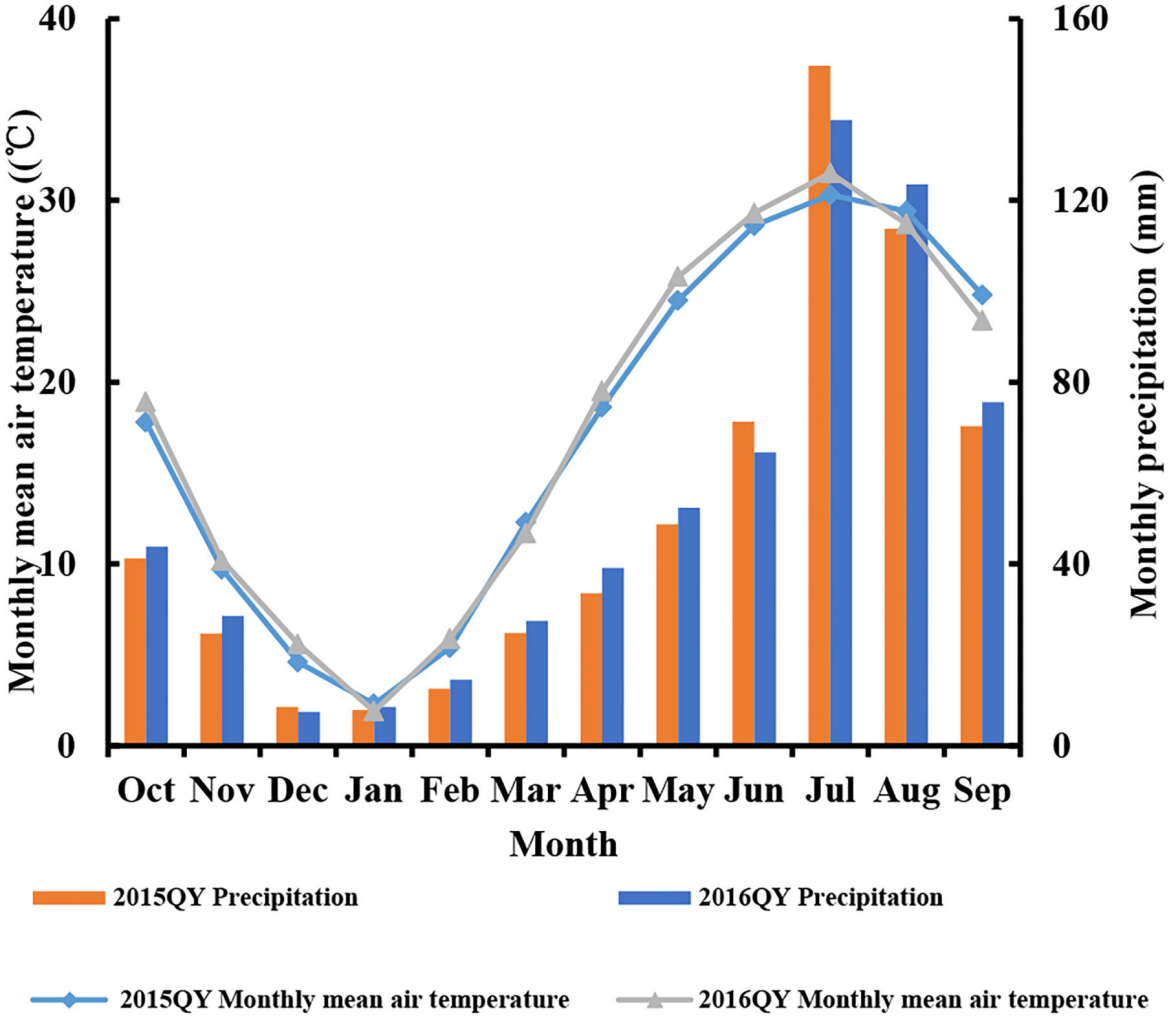
Monthly mean air temperature (°C) and precipitation (mm) during the 2015 and 2016 seasons at Qinyang (QY).

Four field experiments (0–300 kg N ha^−1^) were conducted at Qinyang (35°18′N, and 113°52′) located in China during the 2015 and 2016 seasons (Table 1). The weather condition of the experiment seasons was shown in Figure 1. Before planting, the soil samples (0–20 cm layer) were gathered. The soil type was light loam soil. The total N, Olsen-P, NH_4_OAc-K^+^, and organic matter were measured in the soil samples (Olsen et al., 1954; Bremner and Mulvancy, 1982; Nelson and Sommers, 1982; van Reeuwijk, 1992). The detailed soil physiochemical characteristics (total N, Olsen-P, NH_4_OAc-K^+^, and organic matter) were shown in Table 1. A randomized complete block design was arranged in all experiments with three replications. The ratio of basal fertilizer (before sowing) and top-dressing (V6 stage) of N fertilizer was 50:50%. V6 indicated that the sixth leaf of more than 50% of plants was fully expanded in the field. The triple superphosphate (150 kg P_2_O_5_ ha^−1^) and potassium chloride (120 kg K_2_O ha^−1^) was applied in all the experimental plots as basal fertilizer. The 60 m^2^ size was (10 m length and 6 m width) in each plot from all the experiments. The sowing dates were June 7, June 9, June 10, and June 11 for experiments 1–4, respectively. The cultivars (Dingyou919 and Jundan20) were developed for high-input production systems and were suitable for planting in the experimental region. The planting density of corn was as a stand of 75,000 plants ha^−1^ during the 2015 and 2016 seasons. In total, 60 mm was irrigated after the planting of corn, which can ensure the emergence of corn. Due to the abundant rainfall, irrigation was not needed in the field during corn growth. The weeds, pests, and diseases were controlled using a chemical method.

**Table 1:** Basic information about the four field experiments conducted during the 2015 and 2016 growing seasons at Qinyang.

**Experiment no**.	**Sowing/harvesting date**	**Soil characteristics**	**Cultivar**	***N* (kg N ha^**−1**^)**	**Sampling stage**
Experiment 1	7-June	Type: light loam soil	Dingyou919	N0	V6
(2015)	23-Sept	Organic matter: 11.36 g kg^−1^	(DY919)	N75	V9
Qinyang		Total N: 1.04 g kg^−1^		N150	V12
		Olsen-P: 45.67 mg kg^−1^		N225	
		NH_4_oAc-K^+^: 74 mg kg^−1^		N300	
Experiment 2	9-June	Type: sandy light loam soil	Jundan20	N0	V6
(2015)	26-Sept	Organic matter: 10.83 g kg^−1^	(JD20)	N75	V9
Qinyang		Total N: 0.91 g kg^−1^		N150	V12
		Olsen-P: 35.94 mg kg^−1^		N225	
		NH_4_oAc-K^+^: 67.54 mg kg^−1^		N300	
Experiment 3	10-June	Type: light loam soil	Dingyou919	N0	V6
(2016)	27-Sept	Organic matter: 13.2 g kg^−1^	(DY919)	N90	V9
Qinyang		Total N: 1.12 g kg^−1^		N180	V12
		Olsen-P: 44.56 mg kg^−1^		N270	
		NH_4_oAc-K^+^: 84.56 mg kg^−1^			
Experiment 4	11-June	Type: light loam soil	Jundan20	N0	V6
(2016)	26-Sept	Organic matter: 9.2 g kg^−1^	(JD20)	N90	V9
Qinyang		Total N: 0.87 g kg^−1^		N180	V12
		Olsen-P: 35.87 mg kg^−1^		N270	
		NH_4_oAc-K^+^: 69.45 mg kg^−1^			

### Crop Sampling for Measurements

To obtain a representative plant sample, six plants were randomly and destructively sampled from each plot during the key N top-dressing stages (V6, V9, and V12). V9 and V12 indicated that the ninth and twelfth leaves of more than 50% of plants were fully expanded in the field, respectively. Li-3000 meter was used to measure the green leaf area index (LI-COR, Nebraska, USA). Table 1 showed the sampling stages in each experiment. These samples were collected to determine plant N concentration (PNC) and plant biomass. Plant biomass was obtained by drying plant samples at 80°C to constant weight, followed by analytical balance weighing. These samples were grounded and passed through a sieve (1 mm) for further chemical analysis. The micro-Kjeldahl method was used to determine PNC. The plant N content was determined as the production of plant biomass weight (kg ha^−1^) and PNC on a biomass basis (%). Corn grain yield at maturity was collected from a 12 m^2^ area of each plot by harvesting the whole plant. In total, 14% moisture of grain yield was adjusted.

### Nitrogen Nutrition Index, Nitrogen Deficit Content, and Plant Nitrogen Productivity

The N deficit content of corn was calculated by subtracting plant N_c_ content based on the corn N_c_ curve from actual plant N content under the different N treatments at each sampling date. The crop N_c_ concentration is defined as the minimum N concentration required to obtain maximum crop growth, as proposed for corn (N_c_ = 3.4DM^−0.37^ when DM ≥ 1 t ha^−1^; N_c_ = 3.4% when DM <1 t ha^−1^) by Plénet and Lemaire (2000).


(1) $$\begin{array}{l} N_{\text{cn}}=10N_{\text{c}}\times DM \end{array}$$

(2) $$\begin{array}{l} NDC=N_{\text{cn}}-N_{\text{ac}} \end{array}$$

N_cn_ is a plant critical N content (kg ha^−1^), N_ac_ is the actual plant N content (kg ha^−1^), and 10 is the conversion factor.

The NNI was determined based on plant N_c_ concentration under the different N treatments at each sampling date (Zhao et al., 2017).


(3) $$\begin{array}{l} NNI=N_{\text{a}}/N_{\text{c}} \end{array}$$

N_c_ is the plant N_c_ concentration (%) and N_a_ is the actual PNC (%).

Plant nitrogen productivity (PNP) was defined as the increase in plant biomass per unit of plant N content in this study.


(4) $$\begin{array}{l} PNP=DM/N_{\text{ac}} \end{array}$$

### Photosynthetically Active Radiation

The photosynthetic active radiation of the corn canopy was measured by SUNSCAN Canopy Analysis System (Delta, England). The measurement of PAR was implemented at different stages (V6, V9, and V12) of corn. The measurement was operated at 2 h of intervals from 8:00 a.m. to 4:00 p.m. each day selected. At every measurement period, six points were chosen in every plot. The canopy was averagely divided into 3 leaf layers (upper, middle, and bottom) and measured 4 times from top to bottom at every point. The FIPAR_i_ of every layer was determined as (O'Connell et al., 2004):


(5) $$\begin{array}{l} FIPAR_{\text{i}}=1-PAR_{\text{i}}/PAR_{\text{c}} \end{array}$$

PAR_i_ was the PAR of the i_th_ layer within the canopy (i = 1–3), PAR_c_ was the PAR above 0.2 m of the canopy top. The i_th_ layer within the canopy (i = 1–3) was the upper, middle, and bottom of the canopy.

### Data Analysis

The analysis of variance method was used to analyze the data according to a 2 × 2 × 5 factorial design (Year × Cultivar × N) with three replications (Zhao et al., 2021a,b). The 95% level of significance (least significant difference) was used to assess the difference between treatments (Zhao et al., 2016). The fixed factors were season, cultivar, and N treatments. The SPSS version 13 software package was used to perform all statistical analyses (SPSS Inc., Chicago, USA). Microsoft Excel was used to conduct the regression analysis between NNI, NDC, PNP, and FIPAR (Microsoft Cooperation, Redmond, USA).

## Results

### The Changes in Grain Yield, Plant Biomass, Plant N Content, and Leaf Area Index From V6 to V12 Stages With Cultivar, Year, and N Supply

There were non-significant variations of grain yield between different cultivars and seasons. The plant biomass, plant N content, and leaf area index showed non-significant variations between the different cultivars; however, these differences were significant between the different seasons. The grain yield, plant biomass, plant N content, and leaf area index gradually increased from N0 to N4 treatments. These indices had a significant difference from N0 to N225 treatments in the 2015 season, but there was no significant difference between the high N treatments in the 2015 season (N225 and N300). The grain yield of N4 treatment was 47.1% higher than the yield of N0 treatment (Table 2).

**Table 2:** Grain yield, plant biomass, leaf area index, and plant N content of two corn cultivars at the three growth stages under five N levels during the 2015 and 2016 seasons.

**Treatment**	**Yield (kg ha^**−1**^)**	**Plant biomass (kg ha** ^**−1**^ **)**			**Leaf area index**			**Plant N content (kg ha** ^**−1**^ **)**		
		**V6**	**V9**	**V12**	**V6**	**V9**	**V12**	**V6**	**V9**	**V12**
Cultivars (C)										
DY919	8969.1a	919.8a	2039.2a	3758.9a	0.81a	1.63a	2.58a	24.5a	49.5a	78.5a
JD20	8816.6a	872.2a	1850.8a	3459.4a	0.77a	1.48a	2.49a	23.5a	45.1a	75.9a
Year (Y)										
2015	8621.1a	796.8b	1832.2b	3189.1b	0.65b	1.47b	2.24b	21.31b	44.6b	68.1b
2016	9110.2a	1019.9a	2085.8a	3684.2a	0.89a	1.66a	2.62a	27.34a	50.5a	79.4a
Nitrogen treatment (N)										
N0	5947.7c	584.4d	1416.8c	2309.5d	0.42c	0.96d	1.31d	12.62c	29.18d	39.95d
N75	8389.9b	790.9c	1604.1c	2988.6c	0.64c	1.17d	1.91c	19.37bc	35.61d	57.68c
N150	9512.5ab	999.3b	2076.2b	3817.2b	0.89b	1.68c	2.76b	27.18b	51.07c	83.97b
N225	10546.5a	1115.4a	2517.2a	4203.5a	1.13ab	2.28b	3.41a	34.24ab	69.22b	103.4a
N300	11242.4a	1240.6a	2712.4a	4125.6a	1.33a	2.58a	3.56a	40.56a	78.38a	108.09a
C × Y	NS	NS	NS	NS	NS	NS	NS	NS	NS	NS
C × N	^*^	NS	^*^	NS	NS	^*^	^*^	NS	^*^	^*^
Y × N	^*^	^*^	^*^	^*^	^*^	^*^	^*^	^*^	^*^	^*^
C × Y × N	NS	NS	NS	NS	NS	NS	NS	NS	NS	NS

*NS represents no significance at the 0.05 probability level*.

* *represents significance at the 0.05 probability level*.

There was a non-significant effect of cultivar × year interaction on yield, plant biomass, plant N content, and leaf area index, while year × N interaction revealed the significant effect on yield, plant biomass, plant N content, and leaf area index (*P* < 0.05). The performance of cultivar × N treatment was not uniform at the different growth stages and indices. The year × cultivar × N interaction had not a significant effect on yield, leaf area index, plant biomass, and plant N content (Table 2).

### The Changes in the Nitrogen Nutrition Index and Nitrogen Deficit Content Across Different Environments

**Figure 2:**
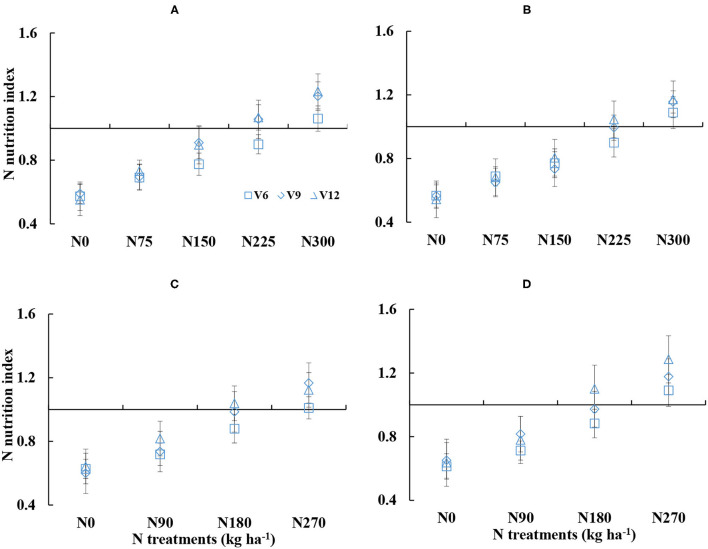
Dynamic changes in the nitrogen nutrition index (NNI) under various nitrogen (N) application rates from V6 to V12 growth stages of corn (**A**: 2015 DY919; **B**: 2015 JD20; **C**: 2016 DY919; **D**: 2016 JD20. V6, V9, and V12 represent the sixth, ninth, and twelfth leaves, respectively).

There were substantial differences in NNI under the different N treatments (Figure 2). The NNI gradually increased with the increased N amounts from V6 to V12 stages of corn. The NNI was ranged from 0.55 to 1.23 and 0.54 to 1.28 for DY919 (Figures 2A,C) and JD20 (Figures 2B,D), respectively, across different N treatments during the 2015 and 2016 growing seasons. There were lower than 1 of NNI values for N0–N150 treatments and for N0–N90 treatments, respectively (plant N status was insufficient), during the 2015 and 2016 seasons. NNI values were near to 1 for N225 and N180 treatments during the 2015 and 2016 growing seasons, which indicated an optimal corn growth (Figure 2). In contrast, there were higher than 1 of NNI values for N300 and N270 treatments during the 2015 and 2016 seasons, respectively (plant N status was sufficient).

**Figure 3:**
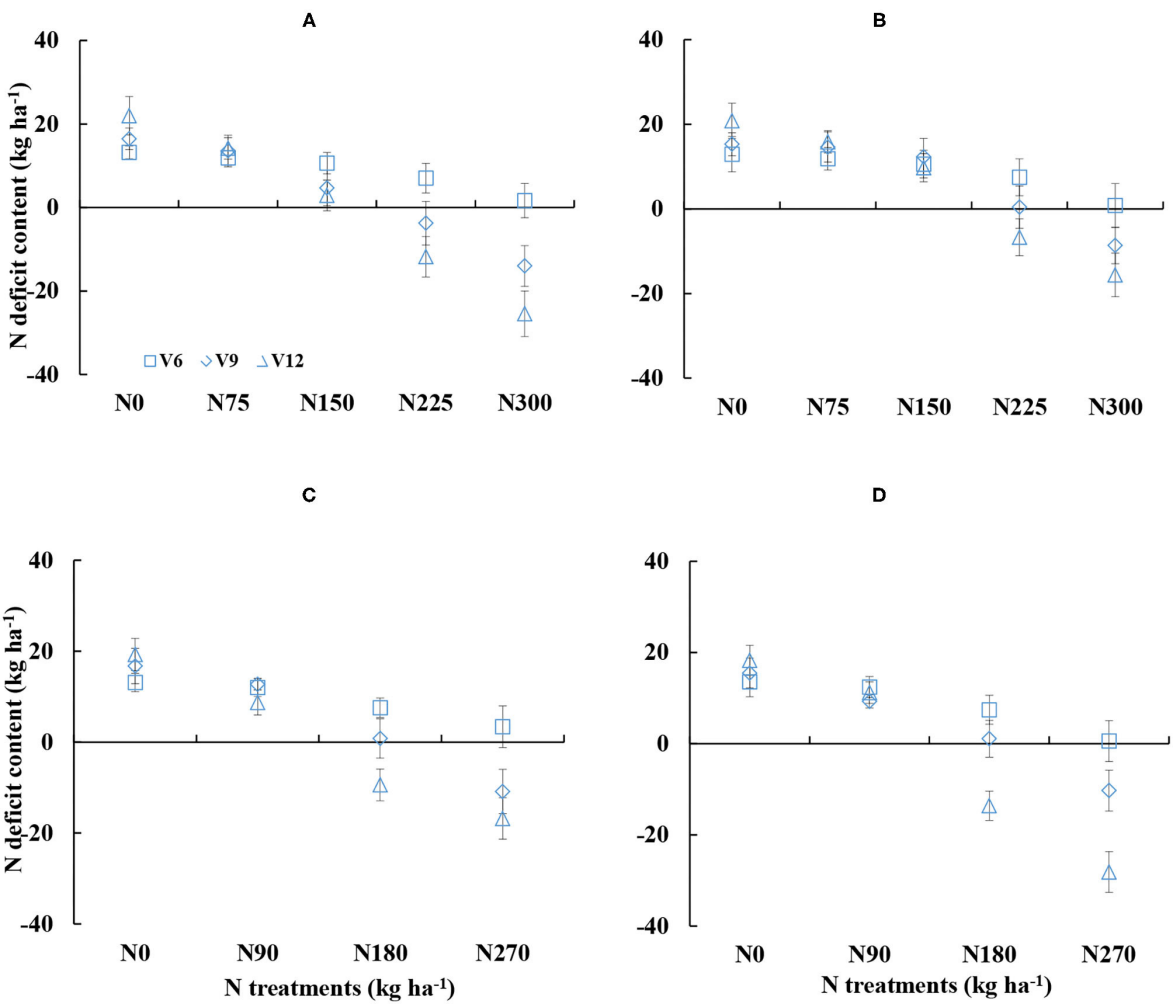
Dynamic changes in the nitrogen deficit content (NDC) under various nitrogen (N) application rates from V6 to V12 growth stages of corn (**A**: 2015 DY919; **B**: 2015 JD20; **C**: 2016 DY919; **D**: 2016 JD20. V6, V9, and V12 represent the sixth, ninth, and twelfth leaves, respectively).

Nitrogen deficit content also showed obvious differences under different N treatments (Figure 3). NDC decreased with increased N supply during corn growth. The NDC values were ranged from −25.48 to 21.99 kg ha^−1^ and from −28.13 to 20.81 kg ha^−1^ for DY919 (Figures 3A,C) and JD20, respectively (Figures 3B,D). NDC values were near to 0 during the 2015 and 2016 growing seasons for N225 and N180 treatments, which indicated an optimal growth of corn (Figure 3). The NDC values were higher than 0 for N0–N150 treatments and for N0–N90 treatments, respectively (plant N status was insufficient). However, during the 2015 and 2016 seasons, NDC values were lower than 0 for N300 and N270 treatments (plant N status was sufficient; refer to Figure 3).

### The Relationship Between Yield and Nitrogen Nutrition Index and Nitrogen Deficit Content

**Figure 4:**
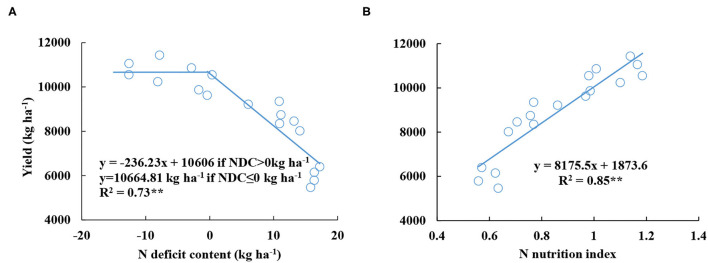
Relationships between grain yield and nitrogen deficit content **(A)** and nitrogen nutrition index **(B)** from V6 to V12 stages during the 2015–2016 growing seasons (experiments 1–4).

The yield had a negative linear-plateau function with the average value of NDC from V6 to V12 stages. The yield decreased with the increasing NDC value until NDC was lower or equal to 0 kg ha^−1^ (Figure 4A). However, the average NNI value had a positive linear relationship with yield (Figure 4B), and the *R*^2^ value (0.83) of the relationship between NNI and yield was higher than the *R*^2^ value (0.73) of the relationship between NDC and yield.

**Figure 5:**
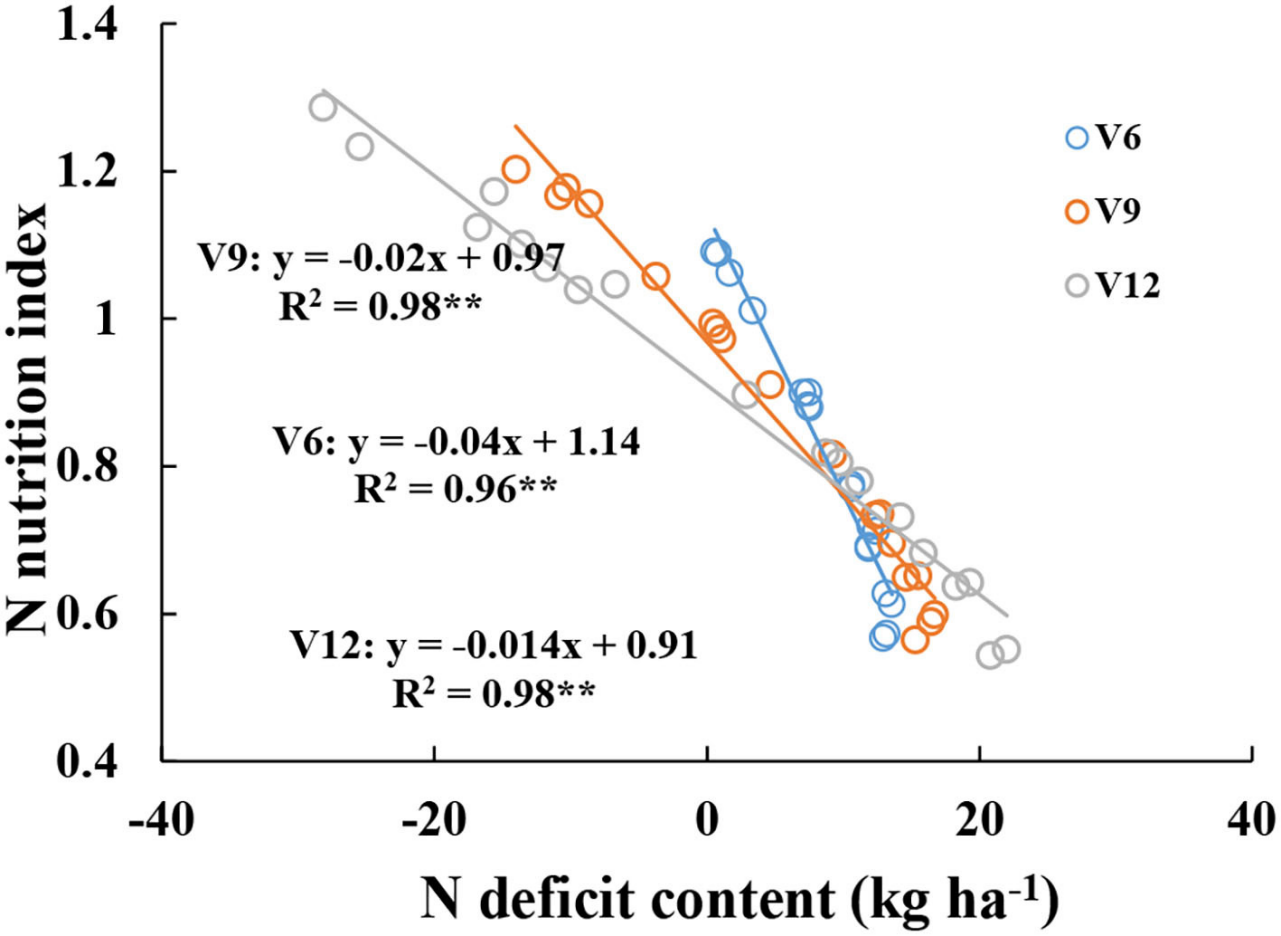
Relationships between nitrogen nutrition index and nitrogen deficit content from V6 to V12 stages of corn during the 2015–2016 growing seasons (experiments 1–4).

There were significantly negative relationships between NNI and NDC from V6 to V12 stages (Figure 5). When NDC was lower or equal to 10 kg ha^−1^, NNI values gradually decreased from V6 to V12 stages at the same NDC value, and when NDC was higher than 10 kg ha^−1^, NNI values gradually increased from V6 to V12 stages at the same NDC value.

### The Changes in PNP From V6 to V12 Stages Across Different Environment

Plant N productivity increased with corn growth. The PNP had an obvious difference between different N treatments, and the PNP gradually decreased with the increased N supply. The maximum value of PNP was 60.46 kg kg^−1^ at N0 treatment of V12 stage in 2015 for DY919. The minimum value of PNP was 30.25 kg kg^−1^ at N300 treatment of V6 stage in 2015 for JD20. The PNP value had a non-significant difference across different years and cultivars. The year × cultivar interaction revealed a non-significant effect on PNP, while cultivar × N and year × N interactions exerted a significant effect on PNP from V6 to V12 stages (*P* < 0.05). There was a non-significant effect of the year × cultivar × N interactions on PNP (Table 3).

**Table 3:** Plant N productivity of two corn cultivars at the three growth stages under five N levels during the 2015 and 2016 seasons.

**Year**	**Cultivar**	**Treatment**	**Plant N productivity (kg kg** ^**−1**^ **)**		
			**V6**	**V9**	**V12**
2015	ZD958	N0	47.58a	48.61a	60.46a
		N75	41.24b	44.56ab	53.23b
		N150	37.71bc	40.97b	48.36c
		N225	34.59c	37.52bc	43.34d
		N300	30.89d	35.06c	38.55e
2015	DH605	N0	47.93a	48.08a	58.13a
		N75	41.37b	45.23ab	52.09b
		N150	37.87bc	43.26b	47.55bc
		N225	33.42cd	38.38c	41.36c
		N300	30.25d	34.57c	37.77d
2016	ZD958	N0	44.44a	49.61a	58.05a
		N90	40.03ab	46.16a	51.8b
		N180	35.68bc	38.76b	44.44c
		N227	32.05c	35.32b	41.94c
2016	DH605	N0	45.21a	48.26a	55.35a
		N90	40.41b	44.02ab	49.75b
		N180	35.64c	39.7b	41.81c
		N227	30.4d	34.33c	36.57c
Y			NS	NS	NS
C			NS	NS	NS
Y × C			NS	NS	NS
Y × N			^*^	^*^	^*^
C × N			^*^	^*^	^*^
C × Y × N			NS	NS	NS

*NS represents no significance at the 0.05 probability level*.

* *represents significance at the 0.05 probability level*.

**Figure 6:**
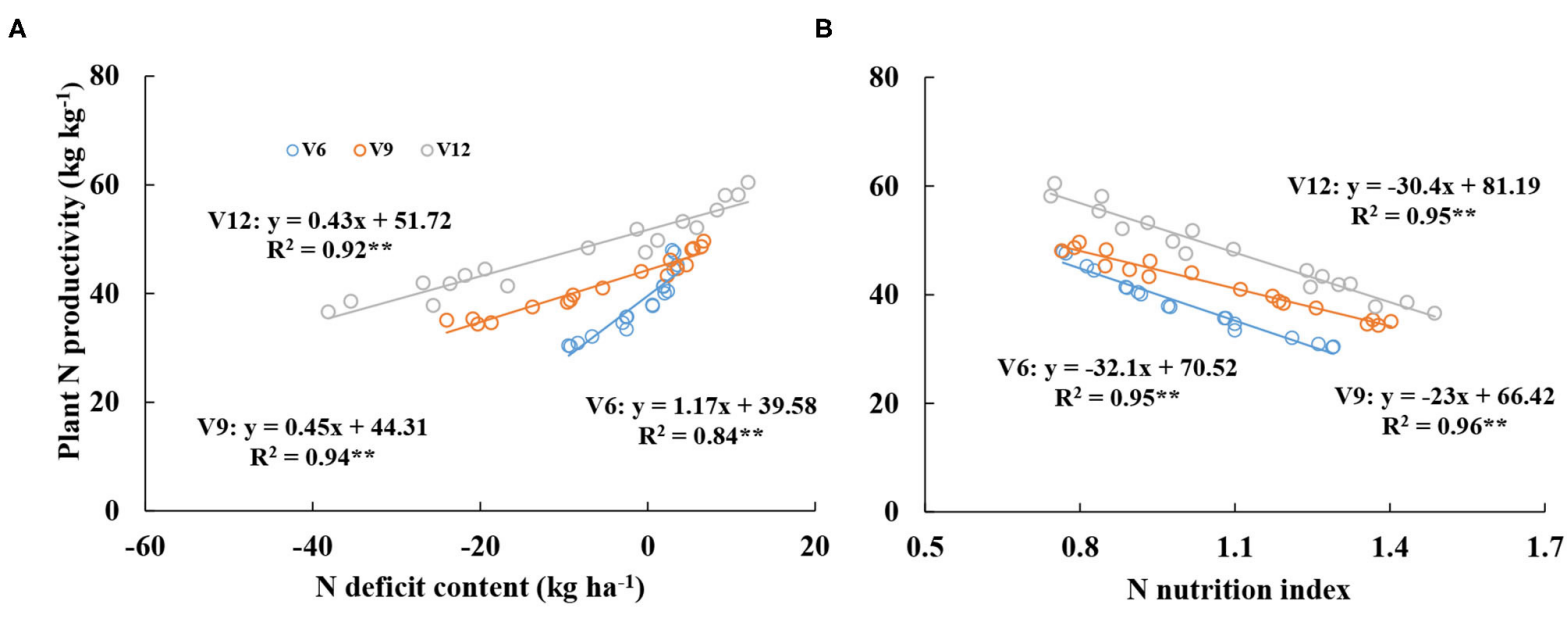
Relationships between plant nitrogen productivity and nitrogen deficit content **(A)** and nitrogen nutrition index **(B)** from V6 to V12 stages of corn during the 2015–2016 growing seasons (experiments 1–4).

Additionally, the relationships between PNP-NNI and PNP-NDC were developed across different growth stages (V6, V9, and V12). Figure 6 showed the significant positive relationship between PNP and NNI, while a significantly negative relationship was observed between PNP and NDC. From V6 to V12 stages, PNP values increased gradually at the same NDC and NNI values. When NDC was equal to 0 kg ha^−1^, PNP values were 39.58, 44.31, and 51.72 kg kg^−1^ at V6, V9, and V12, respectively. In contrast, when NNI was equal to 1, PNP values were 38.42, 43.42, and 50.79 kg kg^−1^ at V6, V9, and V12, respectively.

### The Changes in the Fraction of Intercepted Photosynthetic Active Radiation Across Different Environments

**Figure 7:**
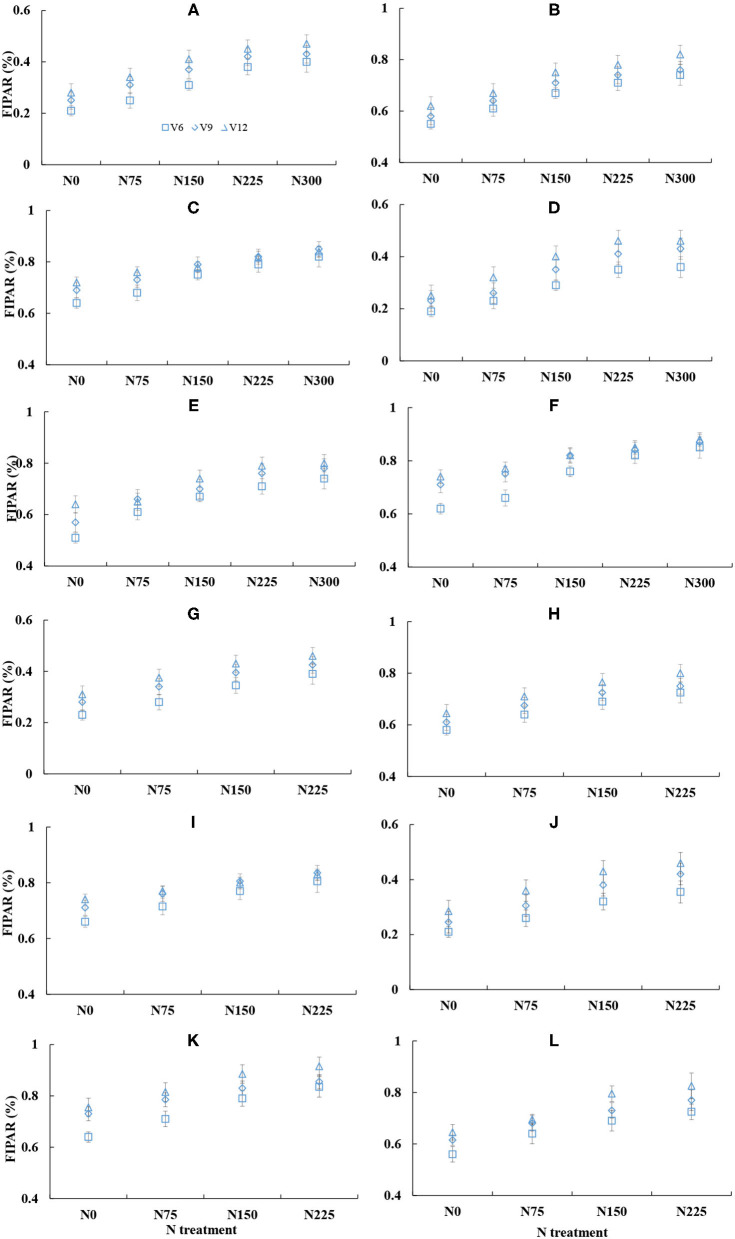
Changes in the fraction of intercepted photosynthetic active radiation (FIPAR) under the different leaf layers across various nitrogen (N) treatment from V6 to V12 stages of corn during the 2015–2016 growing seasons (**A**: 2015 DY919 L1; **B**: 2015 DY919 L2; **C**: 2015 DY919 L3; **D**: 2015 JD20 L1; **E**: 2015 JD20 L2; **F**: 2015 JD20 L3; **G**: 2016 DY919 L1; **H**: 2016 DY919 L2; **I**: 2016 DY919 L3; **J**: 2016 JD20 L1; **K**: 2016 JD20 L2; **L**: 2016 JD20 L3. V6, V9, and V12 represent the sixth, ninth, and twelfth leaves, respectively).

The change of fraction of intercepted PAR was similar across different cultivars and years (Figure 7). The FIPAR within the canopy gradually increased with the growth process of corn (V6 to V12), and the FIPAR values were lower at the lower N treatments. The FIPAR values of the upper leaf layer ranged from 0.2 to 0.4 across different N treatments, while the FIPAR values of the middle and bottom leaf layers were ranged from 0.6 to 0.8. The PAR was mainly intercepted by the upper and middle leaf layers. The FIPAR values of the middle leaf layer were similar to the FIPAR values of the bottom leaf layer, and the FIPAR values of the upper leaf layer were significantly lower than FIPAR values of the middle and bottom leaf layers. The increased proportion of the average FIPAR values of the middle and bottom leaf layers was 18 and 10% at N0 and N225 treatments, respectively. The bottom FIPAR values of the higher N treatments were lower than the bottom FIPAR values of the lower N treatments.

### The Relationship Between the Fraction of Intercepted Photosynthetic Active Radiation From Different Leaf Layers and Nitrogen Nutrition Index and Nitrogen Deficit Content

**Figure 8:**
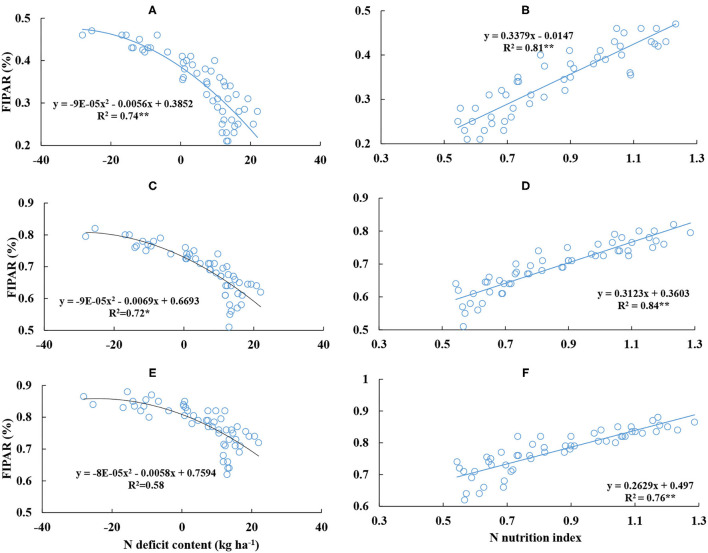
Relationships between the fraction of intercepted photosynthetic active radiation (FIPAR) of different leaf layers and nitrogen deficit content and nitrogen nutrition index from V6 to V12 stages of corn during the 2015–2016 growing seasons (**A,B**: L1; **C,D**: L2; **E,F**: L3).

There was a significantly negative and positive relationship between FIPAR, NDC, and NNI from different leaf layers. The quadratic polynomial could well represent the relationships between FIPAR and NDC from the upper to bottom leaf layers (Figure 8). The maximum *R*^2^ value (0.74) was observed at the upper leaf layer. The linear curve could well represent the relationships between FIPAR and NNI on the canopy scale (Figure 8). The maximum *R*^2^ value (0.84) was observed at the middle level. At the same leaf layer, the *R*^2^ value deduced from NNI was higher than that deduced from NDC. When NNI was equal to 1 and NDC was equal to 0 kg ha^−1^, FIPAR values were 0.32, 0.67, and 0.76 kg kg^−1^ and 0.39, 0.67 and 0.76 kg kg^−1^ at the upper, middle, and bottom leaf layers, respectively.

## Discussion

The N fertilizer had a significantly positive effect on plant biomass, plant N content, leaf area index, and yield of corn. The results of grain yield, plant biomass, plant N content, and leaf area index under different N environments were the same as previous studies on corn (Yue et al., 2012; Zhao et al., 2017). Plant N content was regulated by plant growth rate and soil N availability under the N-limiting environment (Justes et al., 1994). Under a non-limiting N environment, when crop growth rate reaches the maximum value, the plant growth rate remains relatively stable, plant N content would be regulated by soil N availability, which was independent of plant growth rate. The relationships between plant growth rate and plant N content under the N-limiting and non-limiting N environments on corn were developed (Lemaire et al., 2008). The allometric growth relationship between plant N content and plant biomass was shown across different N environments; however, the relationship between plant N content and leaf area index showed linear growth at the same N environment. This difference of the two relationships was based on the decrease of leaf area ratio with the corn growth process. The result of this study further validates the relationship between plant N content and plant growth potential of corn.

Corn N status was diagnosed accurately by NNI and NDC. NNI and NDC values were ranged from 0.54 to 1.28 and from −28.13 to 21.99 kg ha^−1^, respectively. The two indices were significantly affected by the plant growth period, season, cultivar, and N treatment. Similar effects of NDC and NNI of corn have been reported in the other studies (Yue et al., 2012). The change of NNI and NDC were deduced from plant internal N deficit across different N treatments. The plant internal N deficit was calculated from the difference value between plant actual N content and plant N_c_ content, which indicates the trade-off result between plant DM accumulation and external N supply during the vegetative period of corn. The plant N_c_ content was based on N dilution theory. The theory has two main aspects: (i) stem (low N concentration pool) had a greater proportion of biomass and (ii) a decline of leaf N concentration within the canopy for the optimization of plant N allocation (Lemaire et al., 2008). This phenomenon had a close connection with light competition and distribution within the canopy (Lemaire and Gastal, 2009). The interactions of plant growth are related to the perception of the neighboring plants by changing in light spectrum including sensing red: far-red ratio of light by phytochrome and blue light by cryptochrome (Ballaré et al., 1997), which permits plants to forecast the light competition by modifying plant structure (leaf size, tillering, and plant height) and strategies for shade avoidance (Bahmani et al., 2000). This behavior can promote the increased stem proportion, which causes an intensification of plant N dilution process with the crop growth. When light enters into the canopy, the upper leaf layers can intercept most of PAR in the light (Figure 7). The plant will distribute and transport more N into upper leaves that occupy better illuminative conditions for maximum photosynthesis (Hirose and Werger, 1987), the growing leaves at the upper of the canopy usually have the higher N concentration per leaf area (SLN) and photosynthesis rate. The old leaves will remobilize N to new leaves, and their SLN decline as the old leaves are progressively shaded by newer leaves (Lemaire and Gastal, 2009), so the vertical N distribution is not uniform with the plant growth process (Zhao et al., 2018). The difference of the vertical N gradient exists in different crops, which depend on the variable light extinction profile between crops. The vertical N distribution of wheat canopy is steeper than the distribution of corn (Lemaire et al., 2008). Therefore, the plant N dilution is more obvious in wheat than in corn. Due to the clear physiological theory of plant N dilution, the NNI and NDC can well diagnose plant N status quantitatively across different N environments.

Plant N productivity explains the capacity of plant biomass accumulation per plant N content during the crop growth period. PNP value was affected by the crop growth stage, which increased with the growth of corn (Table 3). Our results of PNP were not in line with the result of Greenwood et al. (1991); the PNP remains constant under the N non-limiting condition during the vegetative period of the crop. Under the N non-limiting condition, plant N content of corn increase linearly with the expansion of leaf area, and the rate of plant biomass accumulation per leaf area increases exponentially with the crop growth process (Lemaire et al., 2007). Therefore, the change of PNP is affected by the expansion of leaf area during the corn vegetative stage. When the rate of plant biomass accumulation per leaf area is higher than the rate of plant N uptake per leaf area, the PNP value can gradually increase during corn vegetative growth. On the contrary, plant biomass and leaf area expansion affect the intercepted PAR within the canopy. Leaf area and biomass of corn increase during corn vegetative growth, and the FIPAR performs a corresponding increase. The upper and middle leaf layers can intercept more PAR than the bottom leaf layer within the canopy (Figure 7). The FIPAR values were also affected by plant morphological characteristics (leaf angle and leaf curl degree) and N supply (Bélanger et al., 1992; Bonelli et al., 2016). The reduction of the intercepted PAR is very obvious under the N-limiting condition when the leaf area index is below 3; however, when the leaf area index is above 3, N deficiency would only have a small effect on the intercepted PAR (Bélanger et al., 1992). In this study, the leaf area index was below 3 during V6–V12 stages of corn, and the intercepted PAR was affected by N supply (Figure 8). These results were in agreement with previous studies (Chen et al., 2016).

Previous studies have evaluated the relationships between chlorophyll meter readings, grain yield, grain quality (amylose and protein content), N requirement, crop N partition, photosynthesis, and NNI and NDC (Ata-Ul-Karim et al., 2016a,b; 2017a,b; Hu et al., 2014), which showed a highly satisfactory performance between these relationships. Under the N non-limiting condition, NNI was higher than 1 or NDC was lower than 0 kg ha^−1^ (Figures 1, 2), and plant N nutrition was excessive. The PAR was mainly intercepted by the upper and middle leaf layers, which weaken the light condition of the bottom leaf layer. The photosynthesis ability is lower at the bottom leaf layer, and the respiration of the bottom leaf layer can consume more energy from carbohydrates and proteins in the plant, which is against the plant biomass accumulation (Hikosaka, 2014; Chen et al., 2016). The excessive N was stored in the plant body, which cannot be used to produce more biomass. The excess leaf N accumulation in corn could not increase leaf photosynthetic rate because the excess N in the leaf is channeled into phosphoenolpyruvate carboxylase rather than Rubisco (Uribelarrea et al., 2009). So the PNP value becomes lower with the increase of NNI or the decrease of NDC (Table 3, Figures 6, 8). Under the N limiting condition, NNI was lower than 1 or NDC was higher than 0 kg ha^−1^ (Figures 2, 3), plant N nutrition was insufficient. Leaf area expansion and biomass accumulation are limited under this condition, the capacity of light interception decline at the upper and middle leaf layers, more light can transfer into the bottom of the canopy, therefore, the ability of leaf photosynthesis correspondingly increase at the bottom leaf layer (Hikosaka, 2014; Chen et al., 2016). Plant distribute more N to the bottom leaf layer for metabolism, the absorbed N in the plant body can be more efficiently used to produce biomass, which improves the value of PNP within the total canopy (Table 3). Therefore, NNI had a significantly positive relationship with PNP, and a negative relationship was found between NDC and PNP (Figures 6, 8). Moreover, the *R*^2^ values of the NNI-PNP and NNI-FIPAR relations of different leaf layers were higher than the *R*^2^ values of NDC-PNP and NDC-FIPAR of different leaf layers. The better assessment of plant growth indices (PNP and FIPAR) to N deficiency using NNI than that of NDC assessment was attributed to the calculation method of NNI, which can reduce the effect of the external environment and cultivar characteristic to the regression equations. The NDC value was the actual value of plant N deficit, and it can clearly show the amount of plant internal N deficit under N non-limiting and N limiting treatments. Significantly negative regressions of NDC with NNI from V6 to V12 stages of corn were similar to that of rice and wheat (Ata-Ul-Karim et al., 2013). NNI and NDC have advantages over the other N diagnosis indices as being based on actual crop growth (Ravier et al., 2017). The diagnostic tools based on the N_c_ curve of corn could be used directly for the assessment of plant N status to quantify the response of plant growth to N deficiency. The development of the relationships between NNI-PNP, NNI-FIPAR, NDC-PNP, and NDC-FIPAR was helpful for understanding the change of corn production efficiency and the intercepted PAR across different N treatments at the key top-dressing stage of corn, which can assist in guiding the more reasonable N input to produce the suitable canopy structure and improve N production efficiency for obtaining high corn yield. Further studies are required to test the application of NNI and NDC as an efficient tool for assessing of plant growth under the different N environments.

## Conclusion

The amount of N fertilizer input significantly affected the grain yield, plant biomass, plant N content, and leaf area index. The FIPAR and PNP values were regulated by N fertilizer during the corn vegetative growth. NNI and NDC were calculated based on the N_c_ curve of corn, which can diagnose plant N status and quantify the level of plant N deficit. NNI had a significantly negative relationship with NDC from V6 to V12 stages of corn. The FIPAR and PNP gradually increased with the growth process of corn (V6–V12), and the FIPAR was lower at the N limiting treatments than at the N non-limiting treatments. However, the PNP was higher at the N limiting treatments than at the N non-limiting treatments. NDC and NNI showed strong relationships with PNP and FIPAR. When NDC was equal to 0 kg ha^−1^ or NNI was equal to 1, the PNP was ~39, 44, and 51 kg kg^−1^ across different growth stages, and the FIPAR was ~0.35, 0.67, and 0.76 of the upper, middle, and bottom leaf layers, respectively. This study was conducted to understand the variations in the FIPAR of different leaf layers and PNP under N non-limiting and N limiting conditions at the N key top-dressing stages in corn, which is helpful to develop the feasible canopy structure to improve N fertilizer use efficiency across different N treatment. The newly developed empirical relationships could be affected by the external environment. Therefore, further studies under diverse environmental conditions will be required to check the stability of the newly developed models for their applicability on large scale.

## Data Availability Statement

The original contributions presented in the study are included in the article/supplementary material, further inquiries can be directed to the corresponding authors.

## Author Contributions

BZ and ZL conceived the idea and led the study design. BZ and SA-U-K carried out the experiments, performed the analysis, wrote the manuscript, and edited the manuscript. AD, YG, HL, ZL, AQ, DN, and SM assisted with study design and experiments. All authors contributed to the article and approved the submitted version.

## Funding

This study was supported by the National Natural Science Foundation of China (51609247), Henan Provincial Natural Science Foundation of China (222300420589, 202300410553), the National Public-interested Scientific Institution Based Research Fund of China (FIRI2022-21), the Science and Technology Project of Henan Province (212102110278), Grant-in-Aid for Scientific Research from Japan Society for the Promotion of Science (18KT0087), the China Agriculture Research System (CARS-02), the Agricultural Science and Technology Innovation Program (ASTIP), the National Natural Science Foundation of Henan Province (202300410553), and the Major science and technology projects of Xinxiang City (ZD2020009).

## Conflict of Interest

HL is employed by Henan Weisheng Electric Limited Company. The remaining authors declare that the research was conducted in the absence of any commercial or financial relationships that could be construed as a potential conflict of interest.

## Publisher's Note

All claims expressed in this article are solely those of the authors and do not necessarily represent those of their affiliated organizations, or those of the publisher, the editors and the reviewers. Any product that may be evaluated in this article, or claim that may be made by its manufacturer, is not guaranteed or endorsed by the publisher.
